# Object of Eating

**Published:** 1880-09

**Authors:** 


### OBJECT OF EATING.

Taking food into the body is called eating, passing it from the body is called defecation.

Three fourths of all our ailments occur, or are kept in continuance, by preventing the daily food which is eaten, from passing out of the body, after its substance has been extracted by the living machinery, for the purpose of renovation and growth. A healthy laboring man will eat daily two pounds of solid food, of meat, bread, vegetables and fruit; these two pounds, if brought together in one heap, would fill to overflowing the largest sized dinner plate, aud yet there are myriads of grown-up men and women to whom the idea has never occurred, that if this mass is retained in the body, day by day, inevitable harm must accrue. If a man eats two pounds daily, near two pounds daily must in some way or other pass from his body, or disease and premature death is a speedy and inevitable result.

The object of passing food through the body is threefold in youth; in maturity, two; for growth, sustenance, and repair in the one, in the latter for support and repair only, that is, nutrition; and the process by which the system separates the nutriment from the food is called digestion; the distribution of this digested material to the different parts of the body where needed, for the purpose of being incorporated into bone, flesh, nerve, and tendon, is termed assimilation.

*From "Health and Disease."*

THE condition of the human system most liable to terminate in some incurable disease, is that of habitual "constipation." And still a majority of persons not actively employed suffer from it. It should be known to everybody that "Nelaton's Suppository" will radically cure ninety-nine cases in a hundred. Where costiveness depends upon "piles," the cure is certain; no case of piles will resist this treatment for two days. Price 50 cents a box. Sent free on receipt of price. HALL & RUCKEL, 218 Greenwich Street, New York.

